# Refractory ulcerations associated with livedoid vasculopathy successfully treated with tofacitinib

**DOI:** 10.1111/dth.14470

**Published:** 2020-11-16

**Authors:** Ertao Jia, Guangyao Yan, Min Xiao, Hongling Geng, Jiaxin Wei, Jianyong Zhang

**Affiliations:** ^1^ The Department of Rheumatology the fourth Clinical Medical College of Guangzhou University of Chinese Medicine Shenzhen China; ^2^ The Department of Gynecology Guangdong Provincial Hospital of Chinese Medicine, the Second Affiliated Hospital of Guangzhou University of Chinese Medicine Shenzhen China

Livedoid vasculopathy (LV) is one type of small dermal vessels disorder with chronic pain and intractable ulcers mainly around the malleoli. Previously, microvascular thrombosis was known as the pathogenesis, antiplatelet agents and anticoagulants are the mainstay of treatment.^1^ However, we described a patient with refractory LV, and was successfully treated with tofacitinib. The patient signed a written informed consent form for the purpose of publication of results of this case study.

A 17‐year‐old male presented recurrent ulcers and pain in the maleolli and feet for 3 years. The skin biopsy showed LV because of the presence of vascular wall fibrinoid deposition and vascular lumen fibroid thrombosis in November 2016. The ulcers were recurrent after taking colchicine, thalidomide, dipyridamole, rivaroxaban and aspirin. The ulcers improved after taking methylprednisolone 20 mg per day, however, the ulcers were recurrent as soon as reducing glucocorticoid. There were multiple painful, punched‐out ulcers surrounded by purpuric erythema. Several ulcers healed with white atrophic stellate scars (Figure 1). C‐reactive protein, erythrocyte sedimentation rate, coagulation function, anti‐nuclear antibody, anti‐cardiolipin antibodies, tuberculosis, protein‐C, protein‐S, anti‐thrombin‐III and homocysteine were normal. Factor‐V Leiden mutation was absent. Skin biopsy showed infiltration of lymphocytes around small blood vessels in the dermis (Figure 2), besides vascular wall fibrinoid deposition and vascular lumen fibrinoid thrombosis. Infiltration of lymphocytes suggested inflammation. Tofacitinib 5 mg twice per day was administered, and other drugs were stopped in August 2019. The ulcers were complete healing after 1 month (Figure 3) and there was no pain. The patient took tofacitinib until manuscript submission (September 2020) and the ulcer was no recurrence.

**FIGURE 1:**
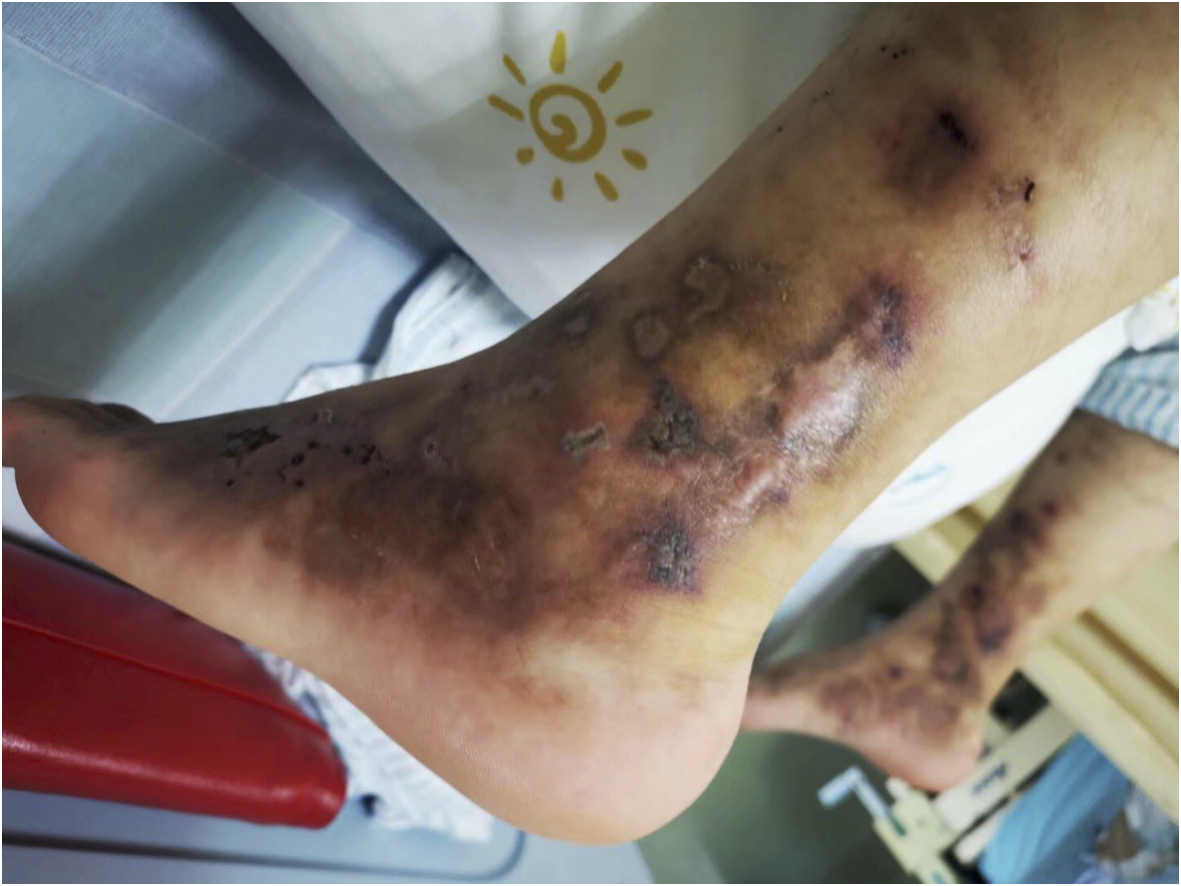
Purpuric macules and livedo racemosa on the dorsal feet and the lower legs before treatment

**FIGURE 2:**
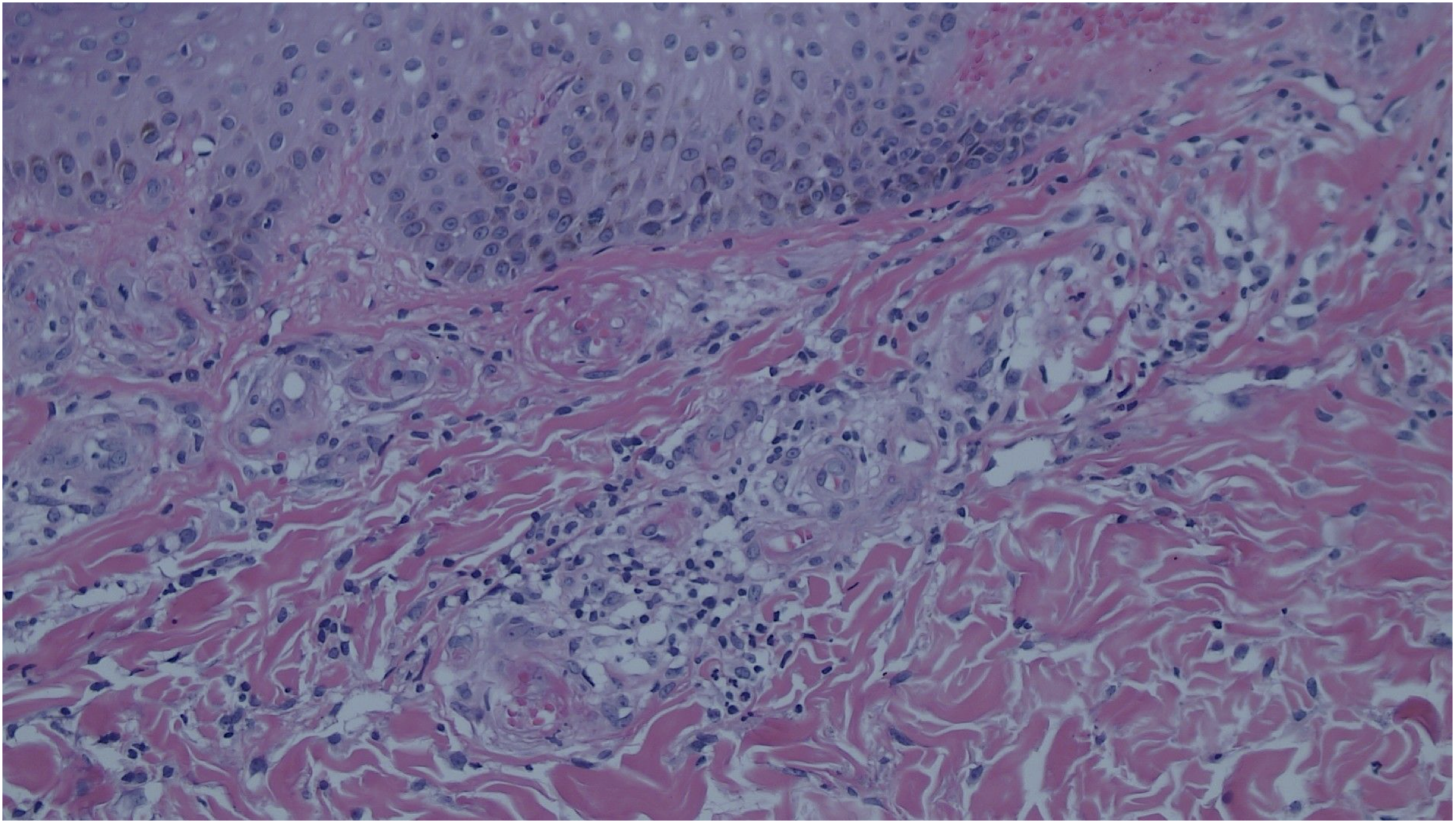
Pathology of skin tissue biopsy, skin biopsy revealed infiltration of lymphocytes around small blood vessels in the dermis, vascular wall fibrinoid deposition and vascular lumen fibrinoid thrombosis (hematoxylin and eosin, original magnification ×100)

**FIGURE 3:**
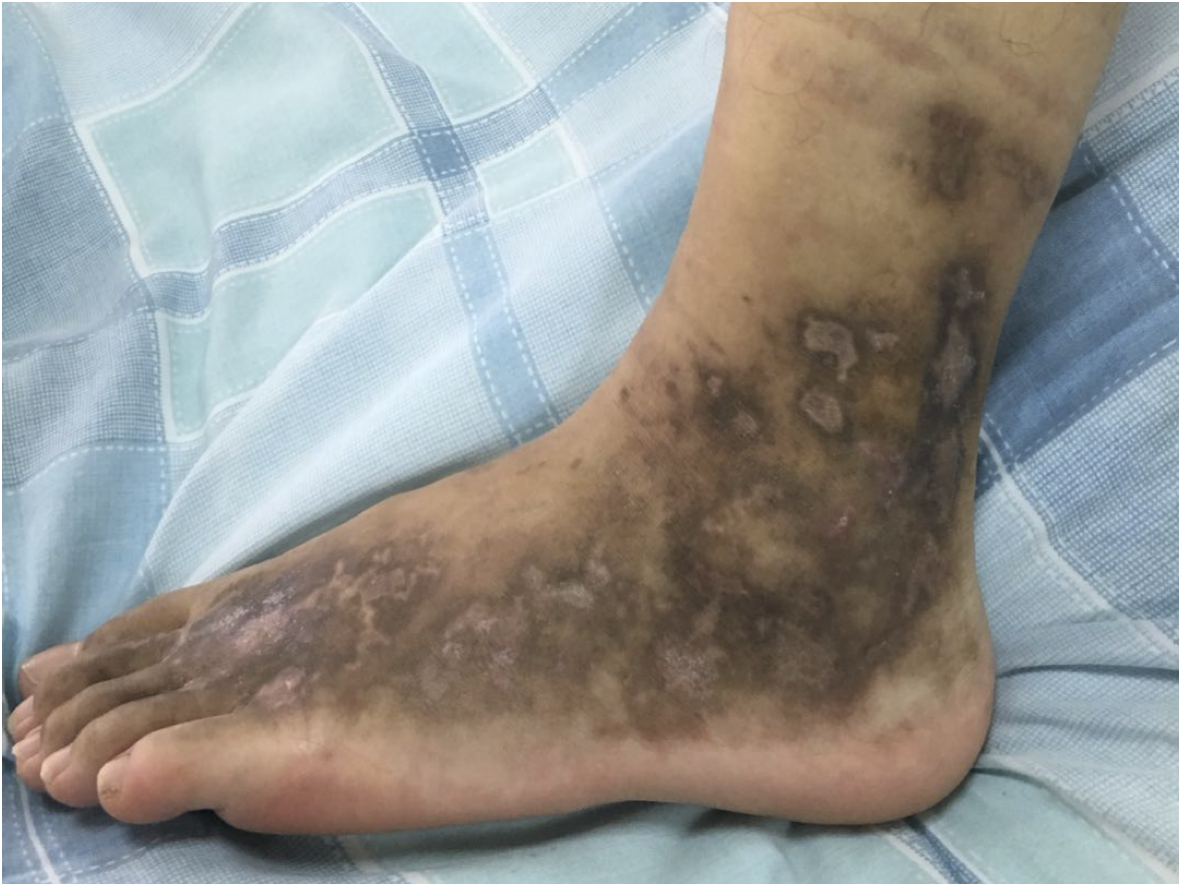
The ulcer improved remarkably after 1 month of treatment

Many studies suggested that vasculitis played an important role in the pathogenesis of LV, and thrombosis was the pathological product of vasculitis. In an observational study, 19 patients (73%) had cutaneous skin pathology with scattered perivascular lymphocytic infiltration in 26 patients.^2^ Kelly et al^3^ reported three cases of lymphocytic thromboarteritis (LTA) that the patients showed a similar manifestations of LV in the late stage of the disease, suggesting a possible etiology of LTA and LV. Irani‐Hakime et al^4^ reported a patient with LV whose skin biopsy showed inflammatory infiltrate with epidermal necrosis. The direct immunofluorescence of blood vessel specimen shows immunoglobulins and complement components in blood vessels on the surface, in the mid‐dermis as well as deep in the dermis.

The successful use of anti‐inflammatory drugs in the treatment of LV also suggests an important role of inflammation. Intravenous immunoglobulins seem to be an effective treatment for patients with refractory LV. At the same time, the success rate of monotherapy of colchicine and prednisolone was higher than that of pentoxifylline and aspirin in patients treated successfully with monotherapy. Successful use of rituximab in three cases and anti‐TNF‐alpha agent in five refractory cases^5^ suggested that the inflammatory pathway may play a role in active vascular disease.

Tofacitinib is a pan‐Janus‐activated kinase (JAK) inhibitor which inhibits vasculitis by regulating T‐cell activation and survival.^6^ T cells depend on signals through their T cell receptor, require input from the cytokine to direct their activation. Cytokine signals trigger the JAK and signal transducer and activator of transcription (STAT) pathway.^7^ Cytokines play a central role in regulating T‐cell activation and survival and exert their effects via JAK3.^8^ It was found that a great enrichment for pathways linked to type I and type II interferons, JAK/STAT and cytokines/chemokines‐related signal in Takayasu's arteritis.^9^ Cytokine signaling dependent on JAK3 and JAK1 is critically important in chronic inflammation of medium, large arteries and Behcet's disease.^6^

We suggest that vasculitis was one of the major pathogenesis of refractory LV. Tofacitinib should be an effective treatment for refractory LV. However, larger or randomized controlled trials were needed.

## CONFLICT OF INTEREST

The authors declare that they have no conflicts of interest.

## AUTHOR CONTRIBUTIONS

**Ertao Jia**, **Guangyao Yan** and **Jiaxin Wei:** Prepared the manuscript. **Jianyong Zhang** conducted the study and revisions from **Min Xiao** and **Hongling Geng**.

## Data Availability

Research data are not shared.
